# Breast cancer survival predicted by *TP53* mutation status differs markedly depending on treatment

**DOI:** 10.1186/s13058-018-1044-5

**Published:** 2018-10-01

**Authors:** Nathan A. Ungerleider, Sonia G. Rao, Ashkan Shahbandi, Douglas Yee, Tianhua Niu, Wesley D. Frey, James G. Jackson

**Affiliations:** 1 Department of Pathology, Tulane School of Medicine, New Orleans, LA USA; 2 Division of Hematology, Oncology and Transplantation, Masonic Cancer Center, University of Minnesota, Minneapolis, MN USA; 3 Department of Biochemistry and Molecular Biology, Tulane School of Medicine, 1430 Tulane Avenue, mail code 8543, New Orleans, LA 70112 USA

**Keywords:** TP53, Breast cancer, Chemotherapy, Hormone therapy, Survival, Senescence

## Abstract

**Background:**

Previous studies on the role of *TP53* mutation in breast cancer treatment response and survival are contradictory and inconclusive, limited by the use of different endpoints to determine clinical significance and by small sample sizes that prohibit stratification by treatment.

**Methods:**

We utilized large datasets to examine overall survival according to *TP53* mutation status in patients across multiple clinical features and treatments.

**Results:**

Confirming other studies, we found that in all patients and in hormone therapy-treated patients, *TP53* wild-type status conferred superior 5-year overall survival, but survival curves crossed at 10 or more years. In contrast, further stratification within the large dataset revealed that in patients receiving chemotherapy and no hormone therapy, wild-type *TP53* status conferred remarkably poor overall survival. This previously unrecognized inferior survival is consistent with p53 inducing arrest/senescence instead of apoptosis. Addition of hormone therapy to chemotherapy improved survival notably in patients with *TP53* wild-type tumors, but not mutant, suggesting hormone therapy could eradicate arrested/senescent cells. Testing this, we found that estrogen receptor-positive, *TP53* wild-type breast cancer cells that were made senescent by doxorubicin treatment were sensitive to tamoxifen.

**Conclusions:**

The poor survival of chemotherapy-treated patients with *TP53* wild-type tumors may be improved by strategies to eliminate senescent cells, including the addition of hormone therapy when appropriate.

**Electronic supplementary material:**

The online version of this article (10.1186/s13058-018-1044-5) contains supplementary material, which is available to authorized users.

## Background

*TP53* is the most frequently mutated gene in cancer, including breast cancer [1]. *TP53* codes for a transcription factor, p53, that initiates transcription of genes involved in cell cycle arrest, cellular senescence, apoptosis, metabolism, DNA repair and other processes following cellular stress [2]. p53 activity is modulated by numerous posttranslational modifications, association with protein partners and regulators, and access to chromatin [2]. When p53 is activated in response to chemotherapy, the constellation of targets transactivated and the degree of induction varies by tumor and cell type, thus altering the fate of the cell [2].

Perhaps owing to the complex molecular activities of p53, there are conflicting reports on its role in breast cancer. *TP53* is mutated in approximately 30% of breast cancers [1], but the clinical relevance of mutant status in a tumor is muddled by different studies that show *TP53* mutation can be detrimental, neutral, or beneficial to outcome (for examples/review, see [3–6]). Comparing these studies to determine why they have reached different conclusions reveals that different methods and surrogates have been used to determine the status of *TP53* in the tumor, and then presence of mutation has been correlated with different clinical endpoints to determine prognostic significance. These include disease-free and overall survival that suggest mutant *TP53* status is detrimental [3, 5], and extent of residual disease present at surgery following neoadjuvant therapy that suggest mutant *TP53* status is beneficial [4, 6]. Problematically, no study has had a sample size sufficient to stratify according to pathological characteristic and/or treatment regimen, potentially obscuring actual differences in survival among populations. Here, we utilize a large dataset with long-term follow-up to define the role of *TP53* mutation in distinct populations of breast cancer patients.

## Methods

### Survival analysis

METABRIC data were accessed through cBioPortal [7] and Kaplan-Meier survival curves with a 20-year endpoint were created (GraphPad Prism, Version 6.07; GraphPad Software, La Jolla, CA, USA) for patients classified as having wild-type (WT) or mutant *TP53*. Statistical significance of overall survival curves was determined using both the Wilcoxon test and log-rank (Mantel-Cox) tests. Multivariate Cox proportional hazards regression models were applied to compute the hazard ratios (HRs) and 95% confidence intervals (CIs) for either *TP53* mutation status or hormone therapy, with control for confounding variables that include tumor histological grade, tumor stage, tumor size, Nottingham Prognostic Index (NPI) [8], estrogen receptor (ER) status, human epidermal growth factor receptor 2 (HER2) status, and progesterone receptor (PR) status, using SAS statistical package (version 9.4; SAS Institute, Inc., Cary, NC, USA). All *p* values were two-tailed. A *p* value < 0.05 was considered statistically significant. Those patients classified as having chemotherapy were treated as follows: DNA damage/metabolism-based chemotherapy, 297 patients [regimens included combinations of cyclophosphamide (C), 5-fluoruracil (F), methotrexate (M), epirubicin (E), adriamycin/anthracycline (A), and capecitabine (cape); CMF: 123, CEF: 1, FAC: 19, ECMF: 70, AC: 69, ACMF: 4, cape: 11]; taxane-based, 6. One hundred four patients were generically noted as “other” or “chemotherapy”. Nothing (e.g., geographic location, year treated, etc.) suggests treatment for these patients was dissimilar to that of the other patients in the study. Thus, the vast majority of patients received DNA-damaging chemotherapy, and very few were treated with taxanes.

### Cell culture

All cell lines were purchased from ATCC (Manassas, VA, USA), and were cultured and MTT assay performed as previously described [9, 10] or according to manufacturer’s instructions. Doxorubicin (Sigma-Aldrich, St. Louis, MO, USA), tamoxifen (Apexbio, Houston, TX, USA), Q-VD-Oph (Apexbio) were used at indicated concentrations.

CRISPR-mediated generation of *TP53* knockout MCF7 cells. *TP53* (5′- CATGTAGTTGTAGTGGATGG-3′) and non-targeting (Rosa26) (5’-CGCCCATCTTCTAGAAAGAC-3′) sense and antisense oligonucleotides with BsmbI-corresponding overhangs (Thermo Fisher Scientific, Waltham, MA, USA) were phosphorylated, heated, then annealed to create double-stranded DNA for inserts that were cloned into BsmbI-cut sites in pLentiCRISPRV2-mcherry (Addgene, Cambridge, MA, USA; #99154). Constructs were confirmed by Sanger sequencing. Lentiviral supernatants from 293 T cells [11] co-transfected with the pLentiCRISPRV2-mCherry vectors, psPAX2 (Addgene# 12260) and pCMV-VSV-G (Addgene# 8454) were used to infect MCF-7 cells by spinfection at 2000 RPM for 20 min. mCherry-positive cells were sorted by Cellular Immunology and Immune Metabolism Core at the Louisiana Cancer Research Consortium, New Orleans, LA, USA. Editing in pools of sorted cells was quantified at approximately 80% by TOPO cloning and Sanger sequencing.

Western blotting was performed as previously shown [10] for p53 (D01, Cell Signaling, Danvers, MA, USA) and actin (BA3R, Thermo Fisher Scientific).

Phase contrast microscope images were captured on an Olympus (Tokyo, Japan) IX71 inverted fluorescence microscope using ×10 objective, and then minimally processed by histogram stretching.

## Results and discussion

**Fig. 1:**
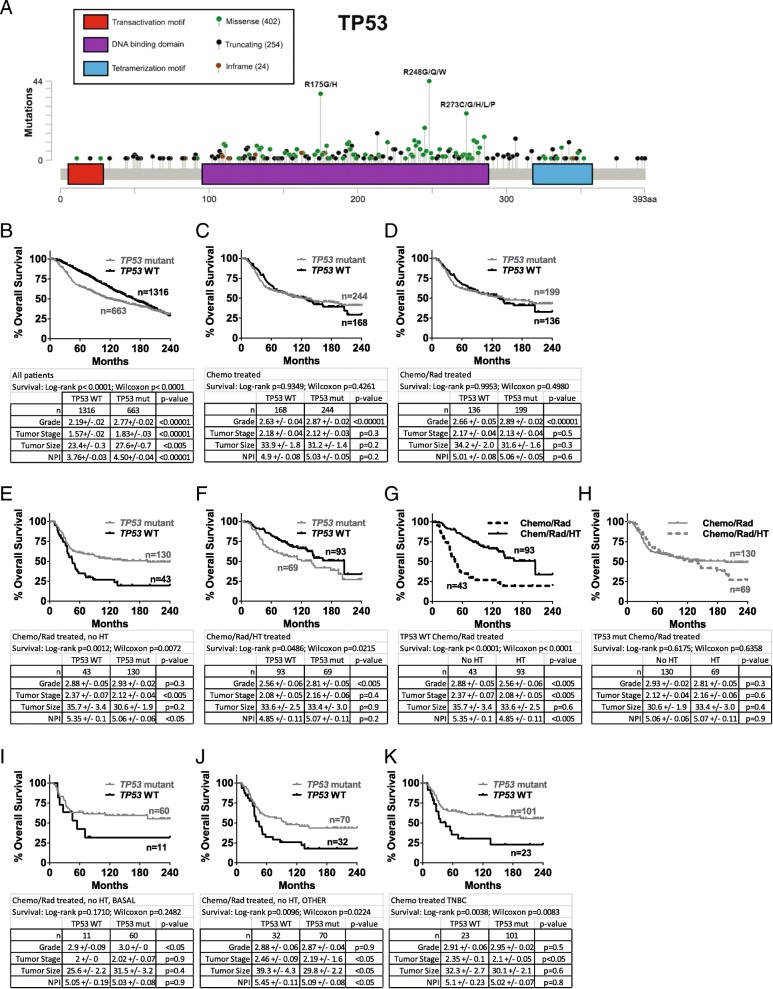
Chemotherapy-treated patients with tumors harboring *TP53* mutation fare equally well or better than patients with *TP53* wild-type tumors. (**a**) Position and frequency of the 663 *TP53* mutations present in the METABRIC dataset accessed through cBioportal. (**b**) Overall survival curves were created for patients in the METABRIC dataset with *TP53* wild-type and mutant tumors from (**b**) all patients; (**c**) those who received chemotherapy (median survival 125 vs 129 months; (**d**) those who received chemotherapy plus radiation (median survival 144 vs 135 months); (**e**) those who received chemotherapy plus radiation but not hormone therapy; (**f**) those who received chemotherapy plus radiation plus hormone therapy. Survival curves were created for patients with *TP53* wild-type (**g**) or mutant (**h**) tumors who received chemotherapy plus radiation and no hormone therapy, or chemotherapy plus radiation plus hormone therapy. Overall survival curves were created for patients with *TP53* wild-type and mutant tumors from (**i**) PAM50 basal-like tumor cohort that received chemotherapy plus radiation but not hormone therapy; (**j**) the other PAM50 classifications combined [claudin low (*n* = 39), HER2 (*n* = 50), luminal A (*n* = 1), luminal B (*n* = 6), normal-like (n = 6)] that received chemotherapy plus radiation but not hormone therapy; (**k**) tumor cohort classified as “triple-negative” in the three gene classifier that received chemotherapy. Statistical differences in survival curves were calculated using both the Wilcoxon test (weighs early events more heavily) and log-rank (Mantel-Cox) tests (weighs events evenly over time). Shown below each survival curve is a table containing the sample size in each arm, the mean +/− standard error of the mean (SEM) and *p* value (unpaired, two-tailed Student’s *t* test) for tumor histological grade, tumor stage, tumor size, and Nottingham Prognostic Index

To overcome the limitations of previous, conflicting studies on the role of *TP53* in breast cancer survival, we analyzed the large METABRIC dataset [12] for overall survival based on *TP53* mutation. Confirming previous studies [3, 5], we found that in all 1979 patients, those with *TP53* mutant tumors (summarized in Fig. 1a) clearly showed a decreased probability of 5-year survival compared to *TP53* WT in a univariate analysis (Fig. 1b) and a multivariate analysis adjusting for covariates that include tumor histological grade, tumor stage, tumor size, Nottingham Prognostic Index (NPI) [8], ER status, HER2 status, and PR status (Additional file 1: Table S1A). However, a trend was evident toward approximately 200 months where the curves appear to cross. This late trend occurred even though *TP53*WT tumors had significantly more favorable grade, stage, size and NPI (Fig. 1b). When patients were stratified by PAM50 subtype, only luminal B and normal-like showed the survival advantage for *TP53* mutant status (Additional file 2: Figure S1A–F).

*TP53* encodes a transcription factor activated by cellular stresses such as DNA damage caused by many commonly used chemotherapy drugs [2]. p53 then initiates transcription of genes involved in cell cycle arrest, cellular senescence, apoptosis, and other processes [2, 11]. *TP53* mutations typically occur in the DNA-binding domain (Fig. 1a), rendering the protein transcriptionally dead, and unable to respond to cellular stress. Because of its prominent role in mediating response to stresses caused by chemotherapy, we next examined the effects of *TP53* mutation on overall survival in various cohorts of treated patients. Survival curves for patients with *TP53*WT and mutant tumors were nearly superimposable for all patients receiving chemotherapy (Fig. 1c), or chemotherapy plus radiation (Fig. 1d). The 60-month advantage in overall survival observed in Fig. 1b for patients with *TP53*WT tumors was no longer evident. Prognostic factors of patients with *TP53* mutant tumors were less favorable or not different than those with *TP53*WT tumors (Fig. 1c, d).

Further examining subsets of chemotherapy-treated patients revealed a remarkable survival advantage in those with *TP53* mutant tumors who did not receive hormone therapy (HT) following chemotherapy and radiation in univariate (Fig. 1e, median overall survival 45 months for *TP53*WT vs 195 months for *TP53* mutant) and multivariate analysis (Additional file 1: Table S1D). Previous studies [5] likely missed this survival benefit due to combining patients who did and did not receive HT (as in Fig. 1c-d). *TP53* mutant tumors were only slightly later stage and higher NPI and no different in tumor grade or size (Fig. 1e). In the cases where HT was added as treatment, the *TP53*WT patients showed a slight survival advantage only in univariate analysis (Fig. 1f, Additional file 1: Table S1E). Unsurprisingly, similar results were found when overall survival was determined based on ER status (Additional file 3: Figure S2A, B), as most patients with ER+ tumors receive HT; and similar trends were observed for all patients receiving chemotherapy, regardless of radiation treatment (Additional file 3: Figure S2C, D).

The entirety of this effect of *TP53*WT changing from unfavorable predictive factor to favorable was due to extension of median lifespan from 45 months to 205 months brought about by addition of HT (Fig. 1g). ER positivity and/or the addition of HT to chemotherapy when patients had *TP53* mutant tumors resulted in no survival benefit (Fig. 1h). Comparing mostly ER+ tumors (those who receive HT) to ER- tumors raises the possibility that ER+ tumors were inherently less aggressive and responded better to chemotherapy plus radiation than ER- tumors, and addition of HT had less of an effect than appears in Fig. 1g. Arguing against this, when *TP53* status was mutant, overall survival was the same for patients with ER- tumors compared to patients with HT-treated, ER+ tumors when both groups were also treated with chemotherapy plus radiation (Fig. 1h). Further, when only grade 3 tumors were evaluated, a similar benefit to HT was observed (Additional file 3: Figure S2E). Lastly, convincing data from early trials show a clear, significant survival benefit in patients with ER+ tumors treated with chemotherapy plus HT when compared to patients with ER+ tumors treated with chemotherapy alone [13, 14].

Triple-negative tumors and those of the basal-like PAM50 classification respond favorably to chemotherapy [4, 15] and also have a high rate of *TP53* mutation [1]. We tested whether the favorable outcome of patients with *TP53* mutant tumors (Fig. 1e) was due to a bias of basal-like subtype in this population. We found that survival curves for patients with *TP53*WT and mutant tumors in PAM50 subtype “basal-like” (Fig. 1i) were very similar to corresponding survival curves of all non-basal subtypes combined (“OTHER”, Fig. 1j) and all the subtypes combined (Fig. 1e). Triple-negative tumors are often basal-like, and again, *TP53* mutant status was highly favorable for patients with triple-negative tumors who received chemotherapy (Fig. 1k, median overall survival 45 months for *TP53*WT vs 263 months for *TP53* mutant [undefined at 240 months]).

**Fig. 2:**
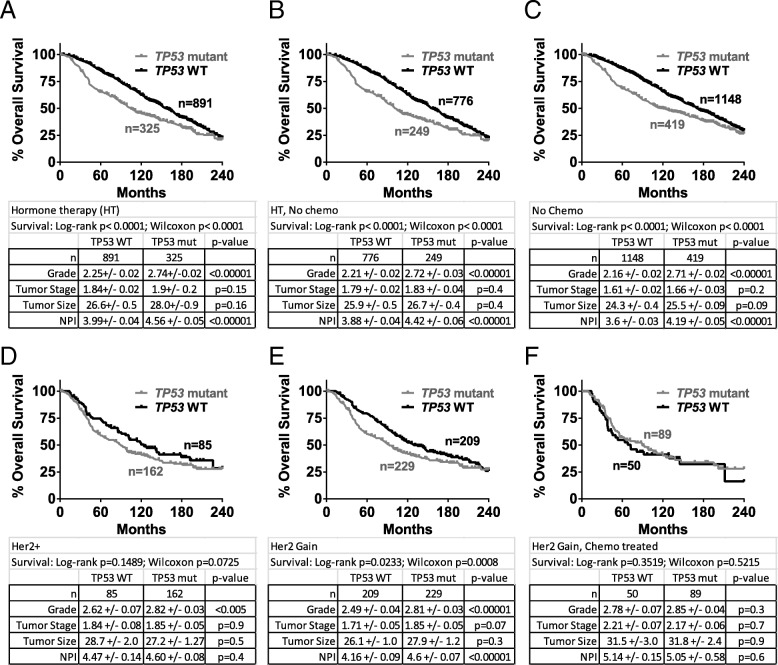
*TP53* mutation portends worse 5-year overall survival for patients who received hormone therapy, and those who received no chemotherapy. Overall survival curves were created for patients with *TP53* wild-type and mutant tumors from cohorts who (**a**) received hormone therapy; (**b**) received hormone therapy but not chemotherapy; (**c**) did not receive chemotherapy. Overall survival curves were created for patients with *TP53* wild-type and mutant tumors from cohorts who (**d**) were HER2+; (**e**) were classified as HER2 gain; (**f**) were classified as HER2 gain and received chemotherapy. Statistical differences in survival curves were calculated using both the Wilcoxon test (weighs early events more heavily) and log-rank (Mantel-Cox) tests (weighs events evenly over time). Shown below each survival curve is a table containing the sample size in each arm, the mean +/− SEM and *p* value (unpaired, two-tailed Student’s *t* test) for tumor histological grade, tumor stage, tumor size, and Nottingham Prognostic Index

We next analyzed data from two more specific breast cancer populations: those treated with HT, and those who are HER2+. In all patients who received adjuvant HT, we found that those with *TP53* mutant tumors had worse 5-year overall survival in univariate and multivariate analyses (Fig. 2a and Additional file 4: Table S2A), as others have observed [3]. Stratification by PAM50 subtype showed this benefit was mostly in luminal B, normal-like and claudin-low subtypes (Additional file 2: Figure S1G–L). In patients who received HT but not chemotherapy and those who did not receive chemotherapy, two cohorts similar to HT, survival trends were similar (Fig. 2b, c and Additional file 4: Table S2B, C). In patients identified as having HER2 gain or HER2 positivity (any treatment), those with *TP53*WT tumors had slightly better overall survival (Fig. 2d, e), but this difference disappeared for patients treated with chemotherapy (Fig. 2f).

**Fig. 3:**
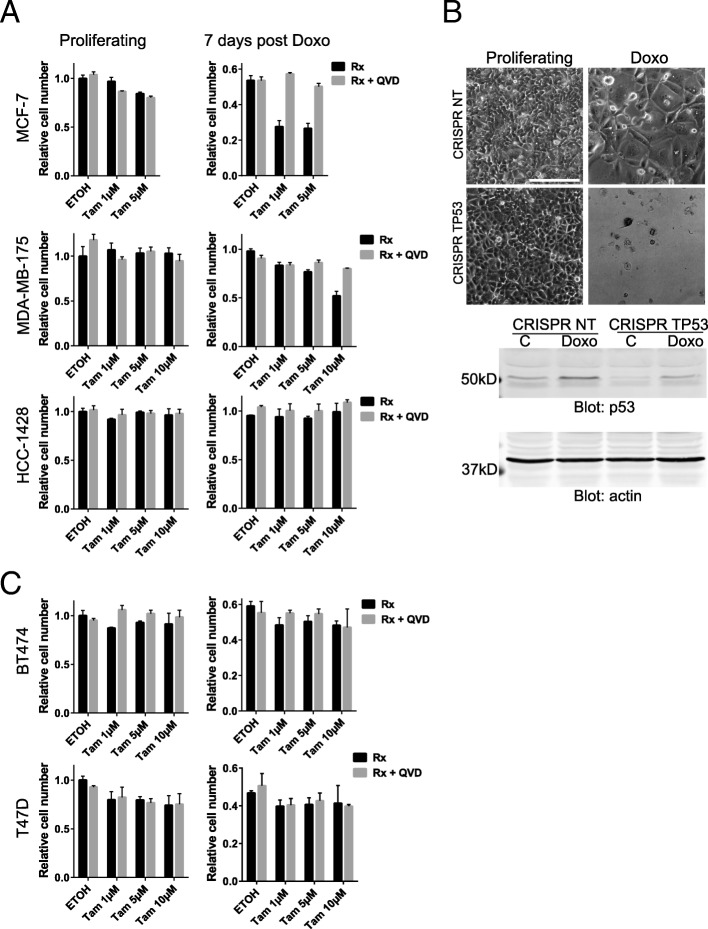
*TP53* wild-type, ER+ breast cancer cells made senescent by chemotherapy are sensitive to tamoxifen. (**a**) *TP53* wild-type, ER+ cells as indicated were plated in triplicate at 80,000 cells per well in a 24-well plate and then treated with 250 nM doxorubicin for 24 h. Seven days later, 1 μM, 5 μM, or 10 μM tamoxifen (Tam) or ethanol vehicle (ETOH) was added as indicated in the figure, with (*gray bars*) or without (*black bars*) the pan-caspase inhibitor QVD. MTT assay was performed 24 h later. Proliferating cells were plated similarly but treated with tamoxifen the next day. (**b**) MCF-7 cells infected using a lentiviral CRISPR Cas9 system with non-targeting (NT) or *TP53* guide RNAs were sorted and then plated and treated with 250 nM doxorubicin as in (**a**). *Upper panel*: light microscopy images were captured for untreated, proliferating cultures or treated cultures as indicated 8 days following treatment. Scale bar is 100 μm. *Lower panels*: western blot for p53 (*upper*) and actin (*lower*). (**c**). *TP53* mutant, ER+ cell lines as indicated were plated, treated, and MTT assay performed as in (**a**). Statistical analyses of these data are shown in Additional file 5: Table S3. Data are representative of at least two independent experiments

The most profound difference observed in our analysis was superior overall survival of patients with *TP53* mutant tumors when treated with chemotherapy plus radiation (Fig. 1e), and the enormous benefit derived from treating *TP53*WT/ER+ patients with HT following chemotherapy plus radiation observed in univariate (Fig. 1g) and multivariate analyses (Additional file 1: Table S1F). These findings are consistent with studies that show p53 preferentially directs a senescence program instead of cell death in *TP53*WT breast cancer [9, 16], and suggest the hypothesis that HT improves survival (Fig. 1g) by eradicating these cells. To test this, we treated ER+, *TP53*WT breast cancer cell lines [17–20] with doxorubicin [9, 21]. MCF-7 and MDA-MB-175 cells were treated with doxorubicin, and 7 days following, addition of tamoxifen resulted in statistically significant loss of viability (at 1 μM and 10 μM, respectively) that was completely rescued by co-treatment with the pan-caspase inhibitor QVD (Fig. 3a, Additional file 5: Table S3). Similarly treated HCC-1428 were insensitive to tamoxifen, and proliferating cell lines were only modestly affected (Fig. 3a). The varying sensitivity of doxorubicin-treated cell lines to tamoxifen is consistent with findings in proliferating cultures [22]. To test if the sensitivity to tamoxifen was dependent on *TP53* status, we first performed a CRISPR Cas9-mediated knockout of *TP53* in the sensitive MCF-7 cells. Consistent with previous findings [9, 23], doxorubicin treatment of control targeted cells resulted in a senescent phenotype with little death, while ablation of p53 resulted in extensive cell death, and no residual cells present for further study (Fig. 3b). Presence of some protein in the *TP53* knockout is consistent with findings that in a fraction of CRISPR targeted *TP53* alleles, a stable protein is made [24]. *TP53* mutant, ER+ cell lines had some surviving cells present after doxorubicin treatment, but these were insensitive to tamoxifen (Fig. 3c). Interestingly, the notion that senescent, *TP53*WT cells, but not *TP53* mutant, are sensitive to HT was supported by the lack of survival benefit to adding HT to chemotherapy in *TP53* mutant tumors that do not undergo senescence (Fig. 1h).

Our findings clarify longstanding conflict in the field about the effect of *TP53* mutation in breast cancer, as we show clear instances of both benefit and harm based on clinical feature/subtype and treatment. Our data suggest previous discordance between chemotherapy studies showing *TP53* mutant tumors respond well initially [4, 6], and survival studies showing *TP53* mutation is harmful [3, 5], is likely because survival analyses failed to separate chemotherapy-treated patients who received HT (Fig. 1f) and those who did not (Fig. 1e).

The data we show do not prove, but are consistent with, induction of a dormant state such as cellular senescence in the *TP53*WT tumors following chemotherapy, which has been previously shown in human patients [16], mouse models [9], and cell lines [9, 21, 10]. Multiple independent studies have shown senescent, dormant cells drive relapse by producing cytokines that promote proliferation, survival, angiogenesis, and an increase in the cancer stem cell population (reviewed in Rao et al. [25]). Thus, finding therapies to eliminate senescent cells in tumors is a promising strategy to improve response. Data presented here suggest one mechanism of action of tamoxifen is to kill senescent cells that persist in *TP53*WT tumors following chemotherapy treatment. Intriguingly, HT did not improve overall survival in patients with ER+, *TP53* mutant tumors that fail to undergo senescence after chemotherapy.

## Conclusions

Chemotherapy-treated patients with *TP53* wild-type tumors have poor survival, consistent with models showing p53 induces cell cycle arrest and senescence instead of cell death. Patient survival in this cohort could be improved by strategies to eliminate senescent tumor cells. One potential mechanism by which HT improves survival is by inducing apoptosis in chemotherapy-induced senescent cells.

## Additional files

Additional file 1: **Table S1.** Multivariate Cox proportional hazards model analysis for Fig. 1b-h. (PDF 401 kb)

Additional file 2: **Figure S1** Overall survival curves by PAM50 classification (for A-F) all patients from METABRIC cohort; (G-L) hormone therapy-treated patients. PAM50 classification was already determined in the METABRIC cohort and accessed through cBioportal. (PDF 1142 kb)

Additional file 3: **Figure S2.** Superior overall survival in patients with ER-negative, *TP53* mutant tumors after chemotherapy-based treatments. Overall survival curves were created for patients with *TP53* wild-type and mutant tumors from cohorts that (A) were ER+ and treated with chemotherapy plus radiation; (B) were ER-negative and treated with chemotherapy plus radiation; (C) received chemotherapy but not hormone therapy; (D) received chemotherapy plus hormone therapy. (E) Overall survival curves were created for patients with histological grade 3, *TP53* wild-type tumors who received chemotherapy plus radiation, or chemotherapy plus radiation plus hormone therapy. Statistical differences in survival curves were calculated using both the Wilcoxon test (weighs early events more heavily) and log-rank (Mantel-Cox) tests (weighs events evenly over time). Shown below each survival curve is a table containing the sample size in each arm, the mean +/− SEM and *p* value (unpaired, two-tailed Student’s *t* test) for tumor histological grade, tumor stage, tumor size, and Nottingham Prognostic Index. (PDF 728 kb)

Additional file 4: **Table S2.** Multivariate Cox proportional hazards model analysis for Fig. 2a-f. (PDF 401 kb)

Additional file 5: **Table S3.** Statistical analyses for Fig. 3. (XLSX 21 kb)

## Abbreviations

ER: Estrogen receptor
HER2: Human epidermal growth factor receptor 2
HT: Hormone therapy
NPI: Nottingham prognostic index
PR: Progesterone receptor
SEM: Standard error of the mean
WT: Wild type
