# Supplementary material for: Breast cancer survival predicted by TP53 mutation status differs markedly depending on treatment
Source: Breast Cancer Res. 2018 Oct 1;20:115. doi: 10.1186/s13058-018-1044-5 (PMC6167800; doi:10.1186/s13058-018-1044-5)
Supplement: Supplementary file 1 — Table S1. Multivariate Cox proportional hazards model analysis for Fig. 1b-h. (PDF 401 kb) [file 13058_2018_1044_MOESM1_ESM.pdf]

A

| Supplemental Table for Fig. 1B | | |
|---|---|---|
| | Hazard Ratio (95% CI) | p-value |
| P53 Status (mutant vs. wild-type) | 1.185 (1.019-1.379) | 0.0275 |
| Tumor Grade (3 vs. ≤ 2) | 1.310 (1.102-1.556) | 0.0021 |
| Tumor Stage (2 vs. 0+1) | 1.070 (0.938-1.220) | 0.3144 |
| Tumor Stage (3+4 vs. 0+1) | 1.222 (0.941-1.586) | 0.1328 |
| Tumor Size | 1.011 (1.007-1.014) | <.0001 |
| Nottingham Prognostic Index | 1.318 (1.217-1.428) | <.0001 |
| ER Status (+ vs. -) | 1.243 (1.031-1.498) | 0.0225 |
| HER2 Status (+ vs. -) | 1.351 (1.126-1.622) | 0.0012 |
| PR Status (+ vs. -) | 0.886 (0.768-1.023) | 0.0984 |

B

| Supplemental Table for Fig. 1C | | |
|---|---|---|
| | Hazard Ratio (95% CI) | p-value |
| P53 Status (mutant vs. wild-type) | 0.805 (0.586-1.105) | 0.179 |
| Tumor Grade (3 vs. ≤ 2) | 1.000 (0.630-1.588) | 0.9985 |
| Tumor Stage (2 vs. 0+1) | 0.695 (0.504-0.959) | 0.0266 |
| Tumor Stage (3+4 vs. 0+1) | 1.085 (0.698-1.687) | 0.7178 |
| Tumor Size | 1.001 (0.995-1.007) | 0.7438 |
| Nottingham Prognostic Index | 1.371 (1.092-1.721) | 0.0065 |
| ER Status (+ vs. -) | 0.880 (0.587-1.319) | 0.5347 |
| HER2 Status (+ vs. -) | 1.391 (1.024-1.888) | 0.0345 |
| PR Status (+ vs. -) | 0.877 (0.582-1.321) | 0.5302 |

C

| Supplemental Table for Fig. 1D | | |
|---|---|---|
| | Hazard Ratio (95% CI) | p-value |
| P53 Status (mutant vs. wild-type) | 0.822 (0.571-1.184) | 0.2922 |
| Tumor Grade (3 vs. ≤ 2) | 0.982 (0.561-1.718) | 0.949 |
| Tumor Stage (2 vs. 0+1) | 0.822 (0.552-1.225) | 0.3356 |
| Tumor Stage (3+4 vs. 0+1) | 1.175 (0.702-1.968) | 0.5388 |
| Tumor Size | 1.001 (0.994-1.008) | 0.8107 |
| Nottingham Prognostic Index | 1.475 (1.130-1.924) | 0.0042 |
| ER Status (+ vs. -) | 0.822 (0.524-1.288) | 0.392 |
| HER2 Status (+ vs. -) | 1.327 (0.929-1.894) | 0.1196 |
| PR Status (+ vs. -) | 0.959 (0.604-1.523) | 0.86 |

D

| Supplemental Table for Fig. 1E | | |
|---|---|---|
| | Hazard Ratio (95% CI) | p-value |
| P53 Status (mutant vs. wild-type) | 0.590 (0.366-0.952) | 0.0308 |
| Tumor Grade (3 vs. ≤ 2) | 1.205 (0.562-2.584) | 0.6324 |
| Tumor Stage (2 vs. 0+1) | 1.214 (0.689-2.138) | 0.5028 |
| Tumor Stage (3+4 vs. 0+1) | 2.398 (1.159-4.960) | 0.0184 |
| Tumor Size | 1.002 (0.993-1.011) | 0.7007 |
| Nottingham Prognostic Index | 1.046 (0.670-1.633) | 0.8447 |
| ER Status (+ vs. -) | 1.094 (0.546-2.190) | 0.7997 |
| HER2 Status (+ vs. -) | 1.736 (1.104-2.731) | 0.017 |
| PR Status (+ vs. -) | 1.095 (0.493-2.430) | 0.8239 |

E

| Supplemental Table for Fig. 1F | | |
|---|---|---|
| | Hazard Ratio (95% CI) | p-value |
| P53 Status (mutant vs. wild-type) | 1.392 (0.823-2.353) | 0.2171 |
| Tumor Grade (3 vs. ≤ 2) | 0.834 (0.392-1.774) | 0.6383 |
| Tumor Stage (2 vs. 0+1) | 0.471 (0.254-0.874) | 0.0169 |
| Tumor Stage (3+4 vs. 0+1) | 0.474 (0.192-1.169) | 0.1051 |
| Tumor Size | 0.999 (0.986-1.011) | 0.8338 |
| Nottingham Prognostic Index | 1.782 (1.250-2.540) | 0.0014 |
| ER Status (+ vs. -) | 1.151 (0.607-2.179) | 0.6669 |
| HER2 Status (+ vs. -) | 0.784 (0.403-1.528) | 0.475 |
| PR Status (+ vs. -) | 0.987 (0.558-1.744) | 0.964 |

F

| Supplemental Table for Fig. 1G | | |
|---|---|---|
| | Hazard Ratio (95% CI) | p-value |
| Hormone Therapy (yes vs. no) | 0.361 (0.200-0.654) | 0.0008 |
| Tumor Grade (3 vs. ≤ 2) | 0.644 (0.272-1.524) | 0.3167 |
| Tumor Stage (2 vs. 0+1) | 0.590 (0.297-1.175) | 0.1335 |
| Tumor Stage (3+4 vs. 0+1) | 0.710 (0.283-1.779) | 0.4642 |
| Tumor Size | 1.002 (0.992-1.013) | 0.6878 |
| Nottingham Prognostic Index | 1.648 (1.055-2.573) | 0.028 |
| ER Status (+ vs. -) | 1.077 (0.540-2.147) | 0.834 |
| HER2 Status (+ vs. -) | 0.917 (0.468-1.798) | 0.8008 |
| PR Status (+ vs. -) | 0.994 (0.536-1.843) | 0.9837 |

G

| Supplemental Table for Fig. 1H | | |
|---|---|---|
| | Hazard Ratio (95% CI) | p-value |
| Hormone Therapy (yes vs. no) | 1.126 (0.718-1.768) | 0.6048 |
| Tumor Grade (3 vs. ≤ 2) | 1.816 (0.894-3.689) | 0.0992 |
| Tumor Stage (2 vs. 0+1) | 0.946 (0.581-1.540) | 0.8235 |
| Tumor Stage (3+4 vs. 0+1) | 1.319 (0.682-2.550) | 0.411 |
| Tumor Size | 1.001 (0.992-1.011) | 0.8289 |
| Nottingham Prognostic Index | 1.423 (1.007-2.010) | 0.0457 |
| ER Status (+ vs. -) | 0.862 (0.460-1.616) | 0.6428 |
| HER2 Status (+ vs. -) | 1.477 (0.966-2.259) | 0.0719 |
| PR Status (+ vs. -) | 0.906 (0.451-1.824) | 0.7829 |
