# Supplementary figures and images for: Breast cancer survival predicted by TP53 mutation status differs markedly depending on treatment
Source: Breast Cancer Res. 2018 Oct 1;20:115. doi: 10.1186/s13058-018-1044-5 (PMC6167800; doi:10.1186/s13058-018-1044-5)

## All patients

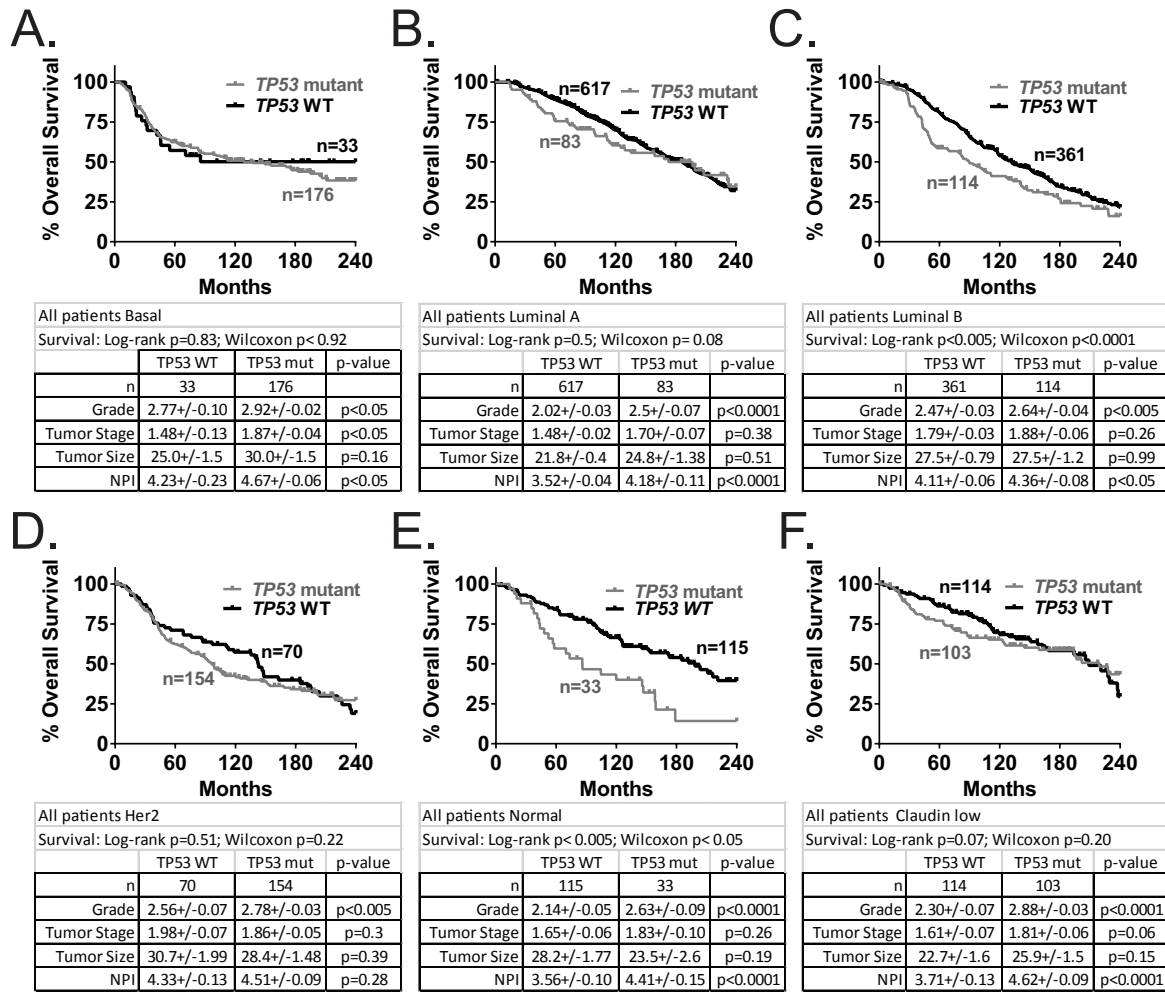

## Hormone therapy Rx patients

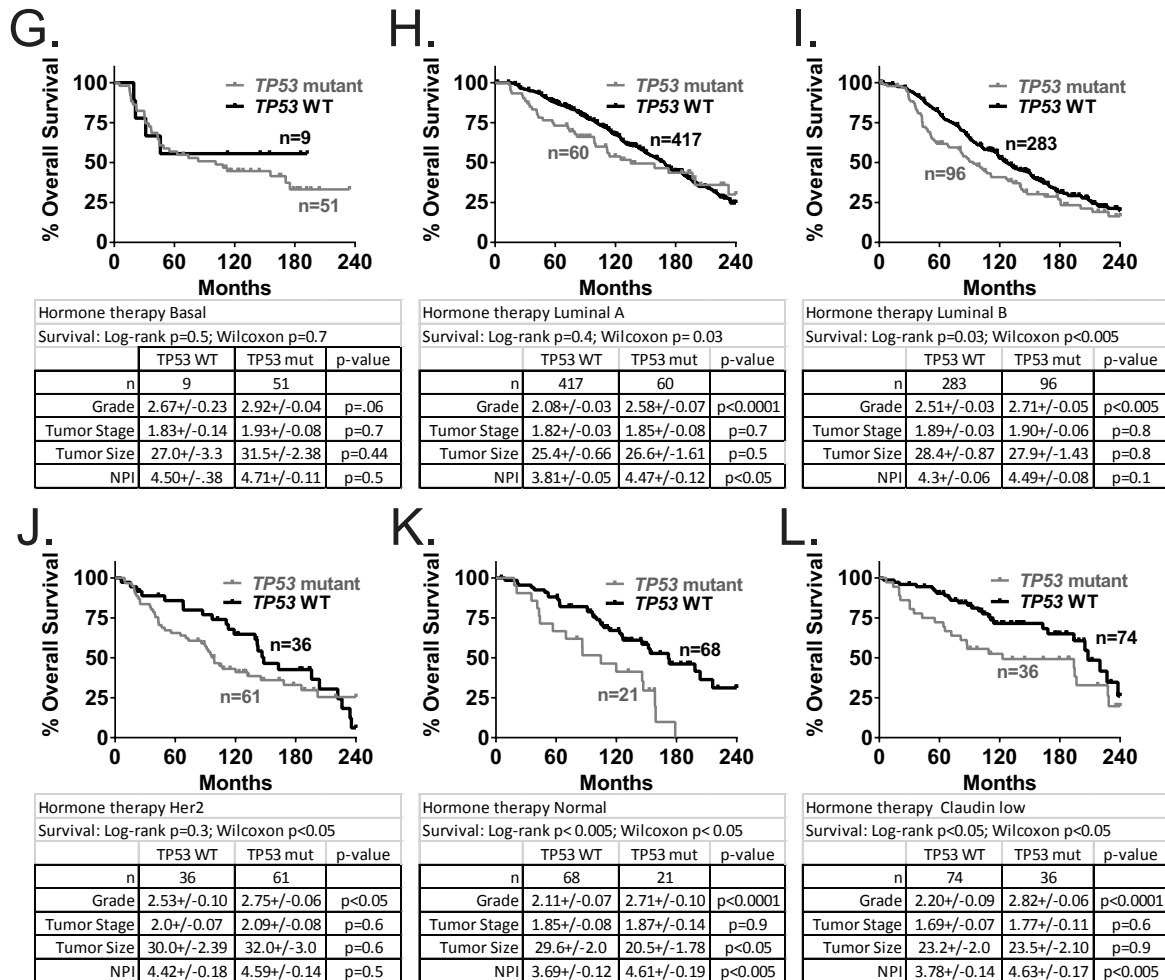

Supplement: Supplementary file 2 — Figure S1 Overall survival curves by PAM50 classification (for A-F) all patients from METABRIC cohort; (G-L) hormone therapy-treated patients. PAM50 classification was already determined in the METABRIC cohort and accessed through cBioportal. (PDF 1142 kb) [file 13058_2018_1044_MOESM2_ESM.pdf]

Figure S2

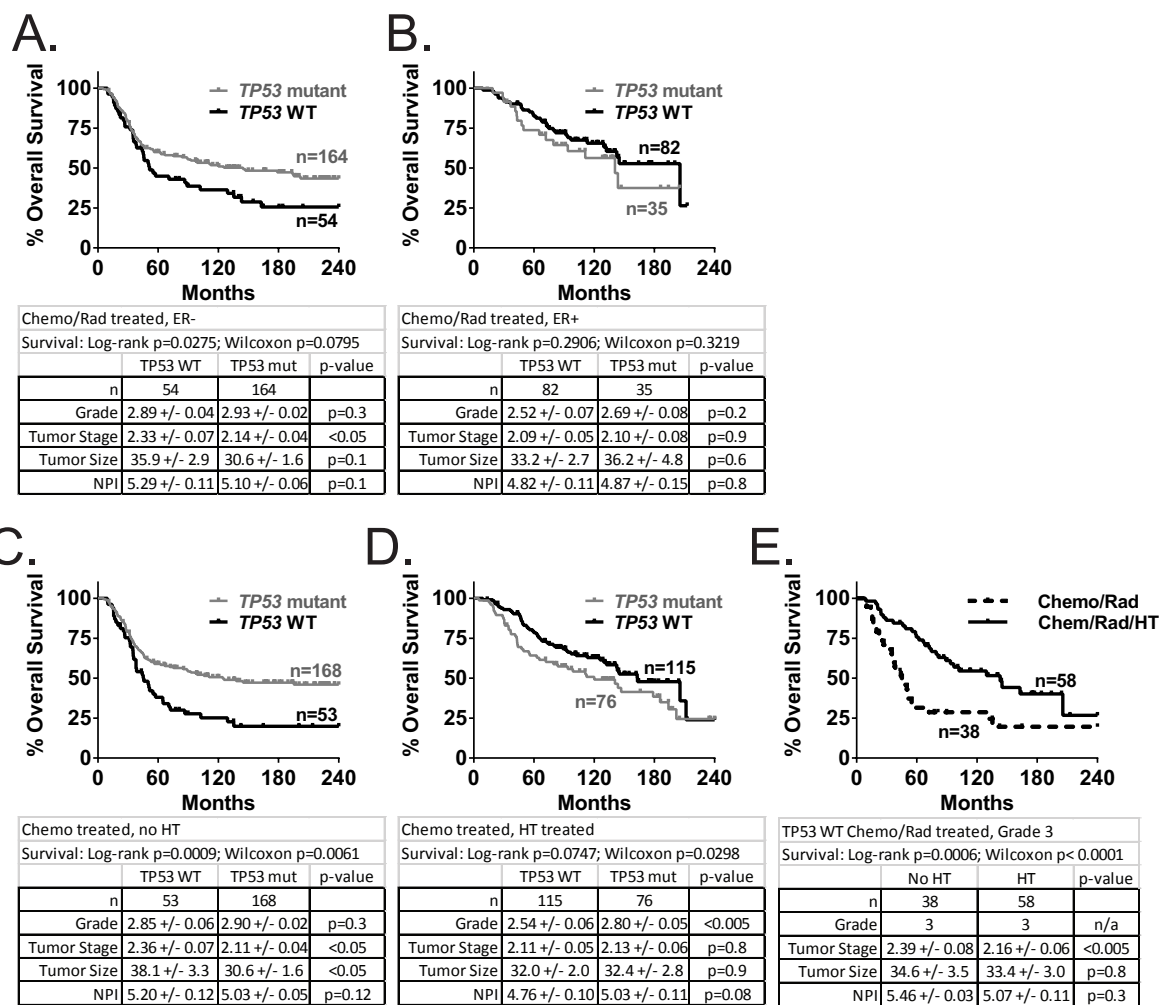

Supplement: Supplementary file 3 — Figure S2. Superior overall survival in patients with ER-negative, TP53 mutant tumors after chemotherapy-based treatments. Overall survival curves were created for patients with TP53 wild-type and mutant tumors from cohorts that (A) were ER+ and treated with chemotherapy plus radiation; (B) were ER-negative and treated with chemotherapy plus radiation; (C) received chemotherapy but not hormone therapy; (D) received chemotherapy plus hormone therapy. (E) Overall survival curves were created for patients with histological grade 3, TP53 wild-type tumors who received chemotherapy plus radiation, or chemotherapy plus radiation plus hormone therapy. Statistical differences in survival curves were calculated using both the Wilcoxon test (weighs early events more heavily) and log-rank (Mantel-Cox) tests (weighs events evenly over time). Shown below each survival curve is a table containing the sample size in each arm, the mean +/− SEM and p value (unpaired, two-tailed Student’s t test) for tumor histological grade, tumor stage, tumor size, and Nottingham Prognostic Index. (PDF 728 kb) [file 13058_2018_1044_MOESM3_ESM.pdf]
