# Supplementary material for: Breast cancer survival predicted by TP53 mutation status differs markedly depending on treatment
Source: Breast Cancer Res. 2018 Oct 1;20:115. doi: 10.1186/s13058-018-1044-5 (PMC6167800; doi:10.1186/s13058-018-1044-5)
Supplement: Supplementary file 4 — Table S2. Multivariate Cox proportional hazards model analysis for Fig. 2a-f. (PDF 401 kb) [file 13058_2018_1044_MOESM4_ESM.pdf]

A

Supplemental Table for Fig. 2A		
	Hazard Ratio (95% CI)	p-value
P53 Status (mutant vs wild type)	1.458 (1.218-1.746)	<.0001
Tumor Grade (3 vs. ≤ 2)	1.389 (1.135-1.701)	0.0014
Tumor Stage (2 vs. 0+1)	0.954 (0.812-1.122)	0.5713
Tumor Stage (3+4 vs. 0+1)	0.910 (0.656-1.264)	0.5752
Tumor Size	1.012 (1.008-1.017)	<.0001
Nottingham Prognostic Index	1.340 (1.215-1.477)	<.0001
ER Status (+ vs. -)	1.226 (0.936-1.606)	0.1381
HER2 Status (+ vs. -)	1.074 (0.830-1.390)	0.5858
PR Status (+ vs. -)	0.884 (0.749-1.044)	0.1471

B

Supplemental Table for Fig. 2B		
	Hazard Ratio (95% CI)	p-value
P53 Status (mutant vs wild type)	1.513 (1.243-1.842)	<.0001
Tumor Grade (3 vs. ≤ 2)	1.461 (1.176-1.814)	0.0006
Tumor Stage (2 vs. 0+1)	1.039 (0.874-1.235)	0.6644
Tumor Stage (3+4 vs. 0+1)	1.015 (0.713-1.444)	0.9359
Tumor Size	1.019 (1.014-1.025)	<.0001
Nottingham Prognostic Index	1.364 (1.228-1.516)	<.0001
ER Status (+ vs. -)	1.069 (0.755-1.514)	0.7075
HER2 Status (+ vs. -)	1.170 (0.879-1.556)	0.2815
PR Status (+ vs. -)	0.909 (0.759-1.089)	0.2999

C

Supplemental Table for Fig. 2C		
	Hazard Ratio (95% CI)	p-value
P53 Status (mutant vs wild type)	1.408 (1.190-1.666)	<.0001
Tumor Grade (3 vs. ≤ 2)	1.458 (1.208-1.760)	<.0001
Tumor Stage (2 vs. 0+1)	1.145 (0.989-1.327)	0.0702
Tumor Stage (3+4 vs. 0+1)	1.122 (0.803-1.567)	0.4999
Tumor Size	1.019 (1.014-1.024)	<.0001
Nottingham Prognostic Index	1.386 (1.267-1.516)	<.0001
ER Status (+ vs. -)	1.196 (0.938-1.525)	0.1492
HER2 Status (+ vs. -)	1.176 (0.930-1.487)	0.1769
PR Status (+ vs. -)	0.888 (0.761-1.037)	0.1333

D

Supplemental Table for Fig. 2D		
	Hazard Ratio (95% CI)	p-value
P53 Status (mutant vs wild type)	1.519 (1.031-2.238)	0.0343
Tumor Grade (3 vs. ≤ 2)	1.543 (0.961-2.479)	0.0728
Tumor Stage (2 vs. 0+1)	1.105 (0.760-1.605)	0.6016
Tumor Stage (3+4 vs. 0+1)	0.926 (0.492-1.742)	0.8112
Tumor Size	1.016 (1.005-1.027)	0.004
Nottingham Prognostic Index	1.480 (1.182-1.854)	0.0006
ER Status (+ vs. -)	1.174 (0.768-1.794)	0.4585
PR Status (+ vs. -)	0.812 (0.502-1.315)	0.398

E

Supplemental Table for Fig. 2E		
	Hazard Ratio (95% CI)	p-value
P53 Status (mutant vs wild type)	1.239 (0.936-1.641)	0.1343
Tumor Grade (3 vs. ≤ 2)	1.384 (0.973-1.967)	0.0703
Tumor Stage (2 vs. 0+1)	1.070 (0.809-1.417)	0.634
Tumor Stage (3+4 vs. 0+1)	0.892 (0.539-1.475)	0.6549
Tumor Size	1.017 (1.009-1.025)	<.0001
Nottingham Prognostic Index	1.360 (1.154-1.604)	0.0002
ER Status (+ vs. -)	0.931 (0.657-1.318)	0.6864
HER2 Status (+ vs. -)	1.111 (0.840-1.471)	0.46
PR Status (+ vs. -)	1.032 (0.750-1.421)	0.8472

F

Supplemental Table for Fig. 2F		
	Hazard Ratio (95% CI)	p-value
P53 Status (mutant vs wild type)	0.731 (0.439-1.217)	0.228
Tumor Grade (3 vs. ≤ 2)	1.107 (0.524-2.337)	0.7899
Tumor Stage (2 vs. 0+1)	0.805 (0.472-1.373)	0.4267
Tumor Stage (3+4 vs. 0+1)	0.656 (0.309-1.394)	0.2731
Tumor Size	1.022 (1.011-1.034)	0.0002
Nottingham Prognostic Index	1.101 (0.747-1.622)	0.6275
ER Status (+ vs. -)	0.712 (0.331-1.532)	0.3847
HER2 Status (+ vs. -)	1.418 (0.788-2.554)	0.2443
PR Status (+ vs. -)	0.968 (0.417-2.247)	0.9403
